# A Soxhlet Extract of *Gongronema latifolium* Retains Moderate Blood Glucose Lowering Effect and Produces Structural Recovery in the Pancreas of STZ-Induced Diabetic Rats

**DOI:** 10.3390/medsci4020009

**Published:** 2016-04-25

**Authors:** Bassel Al-Hindi, Nor A. Yusoff, Item J. Atangwho, Mariam Ahmad, Mohd Z. Asmawi, Mun F. Yam

**Affiliations:** 1 School of Pharmaceutical Sciences, Universiti Sains Malaysia, 11700 Minden, Penang, Malaysia; noradlinyusoff@yahoo.com (N.A.Y.); mariam@usm.my (M.A.); amzaini@usm.my (M.Z.A.); 2 Cluster of Integrative Medicine, Advanced Medical and Dental Institute, 13200 Penang, Malaysia; 3 Department of Biochemistry, College of Medical Sciences, University of Calabar, P. M. B. 1115 Calabar, Nigeria; dratangwho@gmail.com

**Keywords:** soxhlet extractor, *Gongronema latifolium*, diabetes mellitus, hyperglycemia, langerhans islets, streptozotocin, sitostenone

## Abstract

Background: *Gongronema latifolium* Benth. (GL) possesses considerable glucose lowering effects able to be utilized on a large-scale. This paper investigates the effects of a Soxhlet extract on hyperglycemia, Langerhans islets and glucose uptake by abdominal muscles. Methods: Ethanol and a Soxhlet apparatus were used to obtain GL ethanolic Soxhlet extract (GLES). It was then administered to randomly-segregated male *Sprague-Dawley*, normal and STZ-induced diabetic rats, using oral gavage to evaluate blood glucose levels (BGLs), serum lipid profile, insulin levels and the pancreas post-treatment. Results: GLES significantly (*p* < 0.05) decreased BGLs of normal rats in glucose tolerance testing at a dose of 2 g/kg b.w. but failed to do so in diabetic rats undergoing acute 7-h treatment. Given twice-daily, 1 g/kg b.w. of GLES moderately controlled diabetic BGLs starting from day 10. After 14 days of treatment, 1 g/kg and 0.5 g/kg b.w. of GLES caused 44% and 50% respective increases in the average area of Langerhans islets compared to DC. Using isolated rat abdominal muscle, GLES was found to be a mild insulin-sensitizer. GC-MS analysis revealed the presence of the known glucose-lowering phytosterol, Sitostenone. Conclusion: Despite retaining moderate antidiabetic activity, Soxhlet extraction of *Gongronema latifolium* probably leads to the destruction of active heat-liable compounds.

## 1. Introduction

Diabetes Mellitus (DM) is a medical condition marked by hyperglycemia arising from lost control over blood glucose homeostasis. As projected in 2013 by the World Health Organization (WHO), DM is expected to become the seventh leading cause of death in 2030. According to the Malaysian Ministry of Health, there were more than 3,000,000 Malaysians suffering from DM in 2011. This is alarming because the number of DM patients in the Western Pacific Region is expected to double within the next two decades (WHO). Treatment for Type 2 DM, a multifactorial disease, includes many different, expensive combinations of drugs whose pharmacokinetic properties, secondary failure rates and many accompanying side effects often make their continued use problematic [1]. They are associated with weight gain, increased risk of hypoglycemia and increased risk of mortality [2]. This has driven research towards exploring natural alternatives and complementary medicines that have similar capabilities and less adverse effects or costs. Several African and Asian traditional medicinal herbs have been screened and scrutinized [3,4]. In South Africa alone, 28 taxa were found to have reported antidiabetic activity, with more than one of the most popular herbs in ethnomedicine being of the family Apocynaceae [5].

*Gongronema latifolium* Benth. (Apocynaceae) is a perennial edible shrub with a soft stem. The leaves are usually simple, opposite or occasionally whorled with no clear stipules [6]. It is widely used in the West African sub-region for a large number of medicinal and nutritional purposes [7] and to treat a variety of ailments, such as hypertension, diabetes mellitus, malaria, mental and intestinal disorders [8,9]. Traditionally, the leaves of *Gongronema latifolium* (GL) were cooked as a vegetable soup for diabetes [10]; and in some African cultures, it is utilized as a spice to support the pancreas [11]. Early reports [9,12] justified GL’s traditional use because it ameliorated the oxidative stress which accompanied DM and underlined many of the complications of diabetes. Recent reports [7,13,14,15,16] have confirmed the anti-hyperglycemic activity of GL and found it to be superior to that of *Nauclea latifolia* [17] and comparable to that of *Vernonia amygdalina *[18]*.* The extract has also been incorporated in a commercialized antidiabetic product in the United States [7]; and decoctions of the plant have been tested on human subjects [14]. The herb seems to have the prospect of being exploited by the pharmaceutical industry locally, in Nigeria and globally.

Although extraction of an herbal material can be achieved in many ways, such as mechanical pounding, decoction, maceration, pressurized-liquid extraction, microwave-assisted extraction, *etc.* [19], preparing an extract outside the scope of pilot studies often calls for the use of heat [20]. Soxhlet extraction gives greater extraction efficiency than other methods [21]. This study is aimed to evaluate the effect of a Soxhlet leaf ethanolic extract of GL on blood glucose levels upon acute and sub-chronic administration in diabetic rats. The extract‘s effects on serum lipid profile, kidney function indices and insulin-secreting cells (Langerhans islets) were also examined. Furthermore, this work explored the effect of GL on *in vitro* insulin and glucose transduction in isolated rat abdominal muscle and jejunum.

## 2. Materials and Methods

### 2.1. Plant Material

*Gongronema latifolium* Benth. (Apocynaceae), locally known in Nigeria as Utazi or Arokeke, was collected as whole plant from Cross River State at the following GPS coordinates (6°08′17.35″ N 8°41′15.54″ E elev 420 ft) under the supervision of the Department of Biochemistry at the University of Calabar (Calabar Municipal, Nigeria). Authentication was carried out by Pastor Frank, a botanist in the Department of Botany, and voucher specimen (ERU/2011/718) was deposited at the same department. GL leaves were washed and dried in the shade. They were cut into small pieces and ground into fine powder. No special treatment was performed as GL leaves were reported to have synergistic effects amongst their constituents [22]. The powder was properly packaged and arrived within 7 days in the Department of Pharmacology, Universiti Sains Malaysia via courier (Georgetown, Malaysia). Once the powder was received, it was refrigerated at 4 °C until further use.

### 2.2. Preparation of Extract

The dried leaves (~400 g) were placed in a Soxhlet apparatus with ethanol, which was then heated to reflux. The choice of the solvent was to yield the most active extract [23] with documented advantageous properties [9,24]. The heater was set at 60 °C and extraction continued over three days (~50–60 cycles). Anti-pumping silicon granules were added each day before the apparatus was turned on. The extract was filtered and evaporated at 40 °C *in vacuo*. It was then placed in an oven (45 °C) until completely dried. The total dry extract, referred to as GLES, amounted to a yield of ~12% and was refrigerated at 4 °C until further use.

### 2.3. Experimental Animals

Male *Sprague-Dawley* (SD) rats, weighing initially between 180–220 g, were collected from the Animal Research and Service Center, Main Campus, Universiti Sains Malaysia (USM), Penang. Prior to experimentation, the animals were acclimatized to laboratory conditions for one week at the Animal Transit Room, School of Pharmaceutical Sciences, USM. The rats were kept under standard environmental conditions (temp.: 25 ± 5 °C; RH: 50% ± 5%; 12-h light/dark cycle). They had *ad libitum* access to tap water and standard food pellets (carbohydrates 51%; protein 23%; fat 11%; fibers <5%; Ash (minerals) 8%; vitamins <1%) obtained from Gold Coin Feedmills Sdn. Bhd., Butterworth, Penang, Malaysia. The study was approved by the Animal Ethics Committee of the School of Pharmaceutical Sciences, USM (Approval number: USM/Animal Ethics Approval/2013/(90)(509)).

### 2.4. Induction of Diabetes

To induce diabetes in rats, a single intraperitoneal (IP) injection of streptozotocin (STZ) (55 mg/kg) from Sigma-Aldrich (St. Louis, MO, USA) was administered after a 12-h fast. The injected solution had been freshly prepared by reconstituting STZ in cold normal saline. The diabetic condition was confirmed 72 h afterwards by checking the fasting blood glucose (FBG) level using Accu-Chek Performa^®^ glucometer (Roche Diagnostics, Mannheim, Germany). A drop of blood was drawn from a single puncture of the tail vein. Rats with FBG within 15.0–20.0 mmol/L (270–360 mg/dL) were included in the study (approx. 60% of all induced animals).

### 2.5. Experimental Design

#### 2.5.1. Oral Glucose Tolerance Test (OGTT) in Non-Diabetic Rats

After an overnight fast, twenty normal male SD rats were equally divided into four groups (*n* = 5), and FBG was determined. Next, the negative control group (NC) was given 10 mL/kg b.w. of distilled water by oral gavage. The positive control group (PC) received 500 mg/kg b.w. of metformin (Glucophage^®^, Bristol-Myers Squibb, New York, NY, USA) grinded in a suitable amount of distilled water using a mortar and a pestle followed by ultra-sonication (UC-10 Ultrasonic Cleaner, Jeiotech, Seoul, Korea) for 15 min at 37 °C. The extract was reconstituted in distilled water and two different solutions were prepared to enable the administration of 1 and 2 g/kg b.w. of GLES to the two remaining treatment groups, TG1 and TG2, respectively. The volume given *per os* to all animals was similar to the control. Half an hour later, blood glucose levels (BGLs) were determined, just before an oral glucose load (500 mg/kg b.w.) at 0 h. Then, BGLs were measured at 15, 30, 45, 60, 90, and 120 min afterwards. All blood glucose measurements were carried out by using Accu-Chek Performa^®^ (Roche Diagnostics, Mannheim, Germany).

#### 2.5.2. Acute Treatment in STZ-Induced Diabetic Rats

Following a 12-h fast, twenty five diabetic rats were equally divided into five groups (*n* = 5). Additionally, five non-diabetic rats were also fasted and served as the non-diabetic control group. Grouping and treatment illustration can be found in Table 1. All treatments were given by oral gavage and distilled water was used as the solvent. FBG was measured before treatment and BGLs were determined at 1, 2, 3, 5, and 7 h by using Accu-Chek Performa^®^ (Roche Diagnostics, Mannheim, Germany). Blood was obtained via a vein puncture at the tip of the tail.

#### 2.5.3. Sub-Chronic (14 Days) Treatment (Twice-Daily) in STZ-Induced Diabetic Rats

Following a 12-h fast, thirty diabetic rats were equally divided into five groups (*n* = 6). Additionally, six non-diabetic rats were also fasted and served as the non-diabetic control group. Grouping and treatment illustration can be found in Table 2. All treatments were given twice-daily p.o. by using distilled water as the solvent. BGLs were determined on day 1 (before treatment) and days 4, 7, 10, and 15 (after treatment) by using Accu-Chek Performa^®^ (Roche Diagnostics, Mannheim, Germany). Blood was obtained via a vein puncture at the tip of the tail.

#### 2.5.4. Serum Biochemical Parameters

Following the 14-day treatment, the animals were anesthetized and blood was collected via cardiac puncture (~5 mL) into plain tubes. To separate the sera, the samples were allowed to clot for 30 min and were centrifuged at 2500 rpm for 15 min. Separated into two portions, the sera were stored at −20 °C until further use. Lipid profile and kidney function parameters were determined at a local reference laboratory, Gribbles Pathology. Serum lipid profile was determined using ADVIA 2400 Chemistry Analyzer (Siemens, Erlangen, Germany). Kidney function parameters were analyzed by using Olympus AU640 Chemistry Immuno-Analyzer (Olympus, Tokyo, Japan). The concentration of serum insulin was determined by using an ultra-sensitive rat insulin ELISA kit (Crystal Chem Inc., Illinois, IL, USA) with inter- and intra-assay precision of CV ≤10 as per the manufacturer’s handbook.

#### 2.5.5. Histopathological Studies

At the end of sub-chronic treatment, the rats were sacrificed and dissected. The pancreas was removed, weighed and fixed in 10% buffered formalin for seven days. The pancreatic tissue was then dehydrated with a series of alcohols, chloroform and liquid paraffin at 58 °C before being embedded in paraffin. The fixed pancreatic tissues were sectioned (5-micron thickness) and the sections were specifically stained by the Aldehyde Fuschin procedure introduced by Gomori [25]. The stained tissue was evaluated by examination of 10 islets per group by using a Leica MZ6 optical microscope (Leica Microskopie und Systeme, Wetzlar, Germany) equipped with a Leica Qwin (Leica Imaging Systems, Cambridge, England).

### 2.6. Glucose Uptake by Isolated Rat Abdominal Muscle

The uptake of glucose by isolated abdominal muscle was measured according to the method developed by Gray and Flatt [26] with modifications [27,28]. Rats weighing between 180 and 220 g were sacrificed and their abdominal muscles extracted. Each muscle was cut into small squares (90–150 mg), weighed and left in a Krebs-Ringer bicarbonate (KRB) buffer (KRB: 118 mmol NaCl/L, 25 mmol NaHCO_3_/L, 5 mmol KCl/L, 1.28 mmol CaCl_2_/L, 1.2 mmol MgSO_4_/L, and 1.0 mmol KH_2_PO_4_/L) in the presence of 95% O_2_ and 5% CO_2_ at 37 °C. After 10 min, the muscles were transferred to pre-labeled 1.5 mL Eppendorf tubes filled with KRB solution containing 11.1 mmol d-glucose/L. GLES had been added to the tubes (1 mg/mL) in the presence and absence of 1 IU/mL insulin. The final volume was 1 mL. The muscles were aerated for 5 min and incubated for 30 min at 37 °C and then removed. Next, 30 µL from each tube was added to 3 mL of peridocrome (Boehringer, Mannheim, Germany). The final solutions were incubated at 37 °C for 20 min and measured by using a chemistry analyser, Stat Fax 1937 (Awareness Technology Inc., Palm City, FL, USA). Glucose uptake was expressed as mg Absorbed Glucose/g Muscle.

### 2.7. Glucose Absorption via Isolated Rat Jejunum

Rats weighing between 180–220 g were sacrificed and their abdominal walls dissected. The jejunum (20 cm to 50 cm away from the pylorus) was removed and everted according to Wilson and Wiseman [29] as described by Yusoff*, et al.* [30]. Everted jejuna were cut into 5-cm segments and put into an oxygenated Tyrode solution (137 mmol NaCl/L, 2.7 mmol KCl/L, 1.8 mmol CaCl_2_/L, 1.0 mmol MgCl_2_/L, 12.0 mmol NaHCO_3_/L, 0.2 mmol NaH_2_PO_4_/L, and 5.5 mmol D-glucose/L). Each segment was filled with 0.5 mL of Tyrode and tied at both ends to form a sac. Tubes were filled with 15 mL of Tyrode and gassed with 95% O_2_ and 5% CO_2_ at 37 °C. The sacs were incubated inside the tubes for a period of 90 min. An equal number of tubes contained 1 mg/mL Phlorizin (Sigma-Aldrich, Milwaukee, WI, USA), 1 mg/mL Acarbose (Sigma-Aldrich, Milwaukee, WI, USA), and 1 mg/mL of GLES. Tubes with the Tyrode solution alone served as the negative control. The concentration of glucose was measured before and after incubation by using Stat Fax 1937 (Awareness Technology Inc., Palm City, FL, USA). The amount of glucose transported was calculated based on the glucose remaining outside of the sacs. It was expressed as mg Absorbed Glucose/g Tissue.

### 2.8. Gas Chromatography-Mass Spectrometry (GC-MS)

GLES was prepared in a 5 mg/mL solution using absolute ethanol as the solvent (blank). The sample was analyzed using GC Agilent 6890N/95731 equipped with quadrupole Mass Spectrometer. The capillary column used was an Agilent column (HP-5MS: 122-5532) with designated dimensions (30 m × 0.25 mm × 0.25 μm). Helium (99.999%) was used as the carrier gas (mobile phase) with a flow rate of 1.2 mL/min set to split mode. An aliquot of 1 μL of the sample was injected into the column. The injector was pre-heated to 280 °C. GC oven temperature started at 50 °C and rose to 280 °C at the rate of 20 °C/min without holding. Holding was allowed at 280 °C for 20 min. The MS quadrupole and ion source were set at 150 °C and 230 °C, respectively. The mass spectrum of the compounds in the sample was obtained by the standard electron ionization of 70 eV. The detector was operated in scan-mode to detect molecules in the range of 35–650 amu. Analysis time was 33.50 min. Mass spectra were analyzed using the database of the National Institute of Standards and Technology (NIST 02). Chemical structures and information related to molecular formulas, and activities were obtained using Chemspider, the online database of the Royal Society of Chemistry, UK.

### 2.9. Statistical Analysis

Results were expressed as the mean ± SEM. Statistical significance was investigated using version 21 of the IBM-SPSS statistical program (IBM Corp., Armonk, NY, USA). One-way ANOVA was used followed by Dunnett’s Test as a Post Hoc Test. Pre-treatment and post-treatment comparisons were performed using the paired t-test. Differences were considered significant when *p* < 0.05.

## 3. Results

### 3.1. Oral Glucose Tolerance Test (OGTT) in Normal Rats

**Figure 1:**
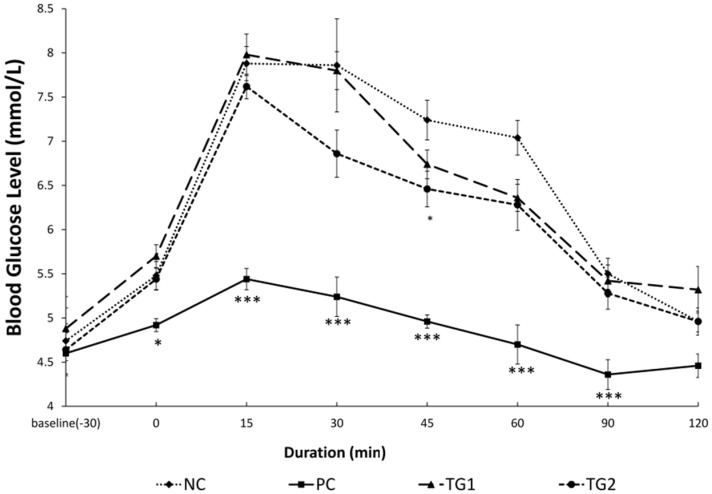
Oral Glucose Tolerance Test with *Gongronema latifolium* extract in normal rats (*n* = 5). Values are expressed as the mean ± SEM, * = *p <* 0.05; *** = *p <* 0.001 *vs.* NC.

In comparison with NC, administration of metformin significantly (*p* < 0.05) reduced BGLs at minute 30 (Figure 1). Furthermore, after glucose loading, PC showed significantly (*p* < 0.001) lower glucose levels at minutes 15, 30, 45, 60, and 90. Similarly, TG2 showed significantly (*p* < 0.05) lower BGLs at minute 45 compared with NC.

### 3.2. Acute (7-h) Treatment in STZ-Induced Diabetic Rats

**Figure 2:**
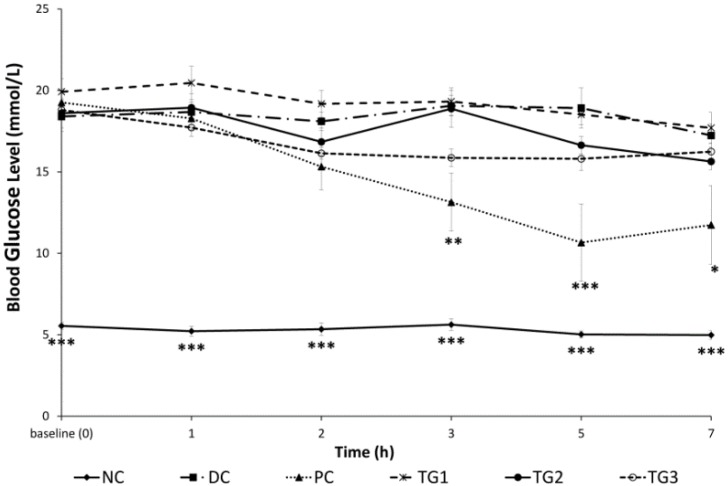
Acute (single) treatment with *Gongronema latifolium* extract in STZ-induced diabetic rats (*n* = 5). Values are expressed as the mean ± SEM, * = *p <* 0.05; ** = *p <* 0.01; *** = *p <* 0.001 *vs.* DC.

Starting from hour 3 post-treatment, PC showed significantly (*p <* 0.01) reduced BGLs (Figure 2). Metformin continued to lower BGLs significantly compared with DC throughout the observation period (*p <* 0.05). GLES administered up to a single dose of 2000 mg/kg did not influence diabetic BGLs. NC showed rather normal glucose levels with the difference in blood glucose remaining as significant (*p <* 0.001) compared with DC throughout the experiment.

### 3.3. Sub-Chronic (14 Days) Treatment in STZ-Induced Diabetic Rats

**Figure 3:**
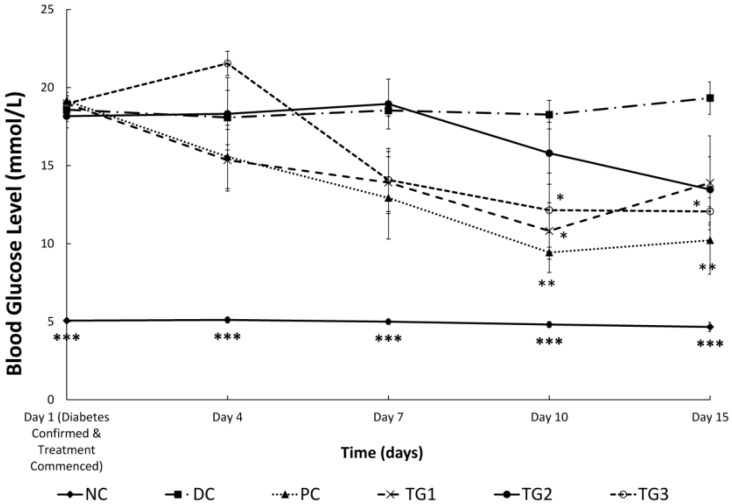
Sub-chronic (14 days) treatment with *Gongronema latifolium* extract in STZ-induced diabetic rats (*n* = 6). Values are expressed as the mean ± SEM, * = *p <* 0.05; ** = *p <* 0.01; *** = *p <* 0.001 *vs.* DC.

**Figure 4:**
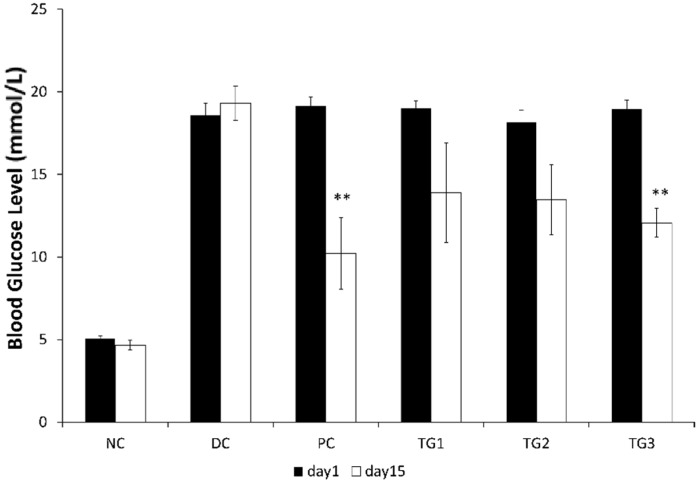
Comparing blood glucose levels before and after 14 days of treatment with *Gongronema latifolium* extract. Values are expressed as the mean ± SEM, ** = *p <* 0.01.

When diabetic SD rats were treated twice-daily for 14 days with GLES, the effect was comparable, yet inferior to metformin: Both metformin (500 mg/kg) and GLES (1000 mg/kg) ameliorated hyperglycemia and induced significantly (*p <* 0.05) lower BGLs starting from day 10 (Figure 3). A transient significant reduction of BGLs was also observed in TG1. Comparison of BGLs before and after treatment showed that when given twice-daily, 1 g/kg b.w. of GLES lowered BGLs as efficiently as 500 mg/kg b.w. of the conventional medicine, metformin (*p <* 0.01) (Figure 4).

#### 3.3.1. *Gongronema latifolium* Subchronic (14 Days) Treatment Effects on Body Weight and Food Intake (kcal/g b.w./day)

**Figure 5:**
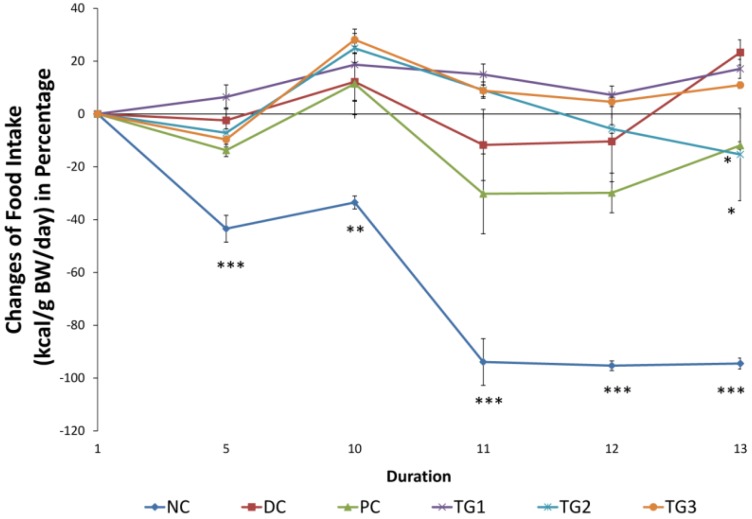
Percentage of change in food intake (kcal/g b.w./day) during sub-chronic treatment with *Gongronema latifolium* extract. Values are expressed as the mean ± SEM, * = *p <* 0.05; ** = *p <* 0.01; *** = *p <* 0.001 *vs.* DC.

Weight changes were monitored throughout the period of the experiment. The increase in the weight of the normal rats was significant (*p* < 0.001) compared to the diabetic control group starting from day 6 until the end of the study. On the other hand, when compared to DC, GLES caused no significant changes in body weight. Initial levels of food consumption were also measured at the beginning of the 14-day treatment and on days 5, 10, 11, 12 and 13. Food intake was calculated based on the feed’s metabolizable energy value as provided by the supplier. It was presented as kcal/g b.w./day (Figure 5). Compared to DC, the normal rats maintained a significantly lower energy consumption (food intake) rate. However, except for day 13, changes of food intake of the extract-treated groups, TG1, TG2, and TG3 showed no significant differences compared to DC. Probably signaling better glycemic control, food intake tended to decrease with the administration of metformin (PC) after day 10. The difference was significant on day 13 compared with DC (*p <* 0.05).

#### 3.3.2. *Gongronema latifolium* Subchronic (14 Days) Treatment Effects on Serum Biochemical Parameters

Compared to the diabetic control (DC) after 14 days of treatment, GLES (1 g/kg) increased HDL significantly (*p <* 0.001). TG3 total cholesterol and HDL levels were significantly (*p <* 0.001) higher compared with NC. Additionally, both TG2 and TG3 showed lower blood Triglyceride levels in a non-significant manner. No observable change in the LDL levels was recorded in the treated groups. On the other hand, signaling an impaired renal function, urea was significantly (*p <* 0.001) higher in all of the diabetic groups as compared to NC. The findings indicated that consecutive administration of GLES to diabetic rats in doses up to 1000 mg/kg twice-daily for 14 days did not affect the levels of blood electrolytes, creatinine, or uric acid. Those findings might be in support of [13] in terms of GL renoprotective properties in diabetes.

#### 3.3.3. Serum Insulin Measurement

**Figure 6:**
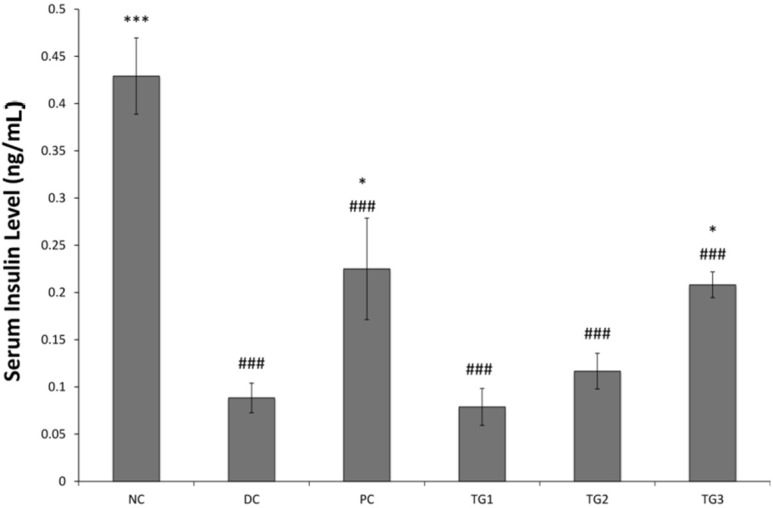
Serum Insulin Levels after 14 days of treatment with *Gongronema latifolium* extract. Values are expressed as the mean ± SEM, * = *p <* 0.05; *** = *p <* 0.001 *vs.* DC; ### = *p <* 0.001 *vs.* NC.

Comparison between NC and DC showed that administration of streptozotocin significantly reduced the level of circulating insulin in the blood (*p <* 0.001) (Figure 6). This highlighted the damage to pancreatic β-cells. However, treatment with metformin (500 mg/kg) and GLES (1000 mg/kg) for 14 days resulted in a significant (*p* < 0.05) positive impact on insulin levels, probably due to marked reduction of BGLs and improvement in animals’ welfare. This is in contrast with *Coscinium fenestratum *of the traditional Ayurveda and Siddha systems of medicine, whose use in STZ-induced animals lowered blood glucose levels but had no impact on serum insulin levels [31]. The effects observed here corresponded with further findings that indicated a structural recovery in Langerhans islets following GLES sub-chronic use.

#### 3.3.4. Assessment of the Area of Pancreatic β-cell-Containing Langerhans Islets

**Figure 7:**
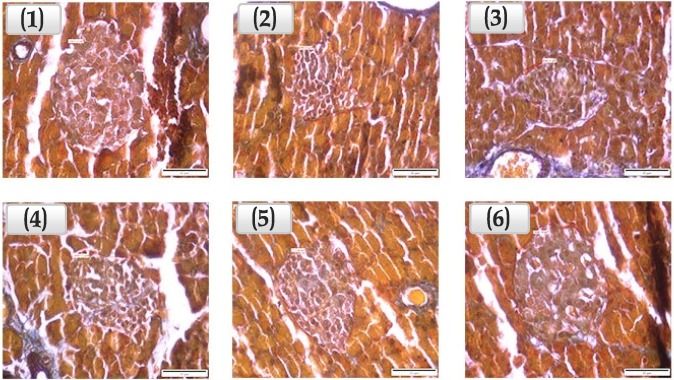
Langerhans islets under 400× magnification power. (**1**) NC: langerhans islets are clearly defined in an organized cyto-architecture. Secretory acini, centroacinar cells and excretory ducts appear to be well defined; (**2**) DC: Cytotoxic effects of Streptozotocin seem to have caused a distortion in the architecture of the pancreatic tissues. The islets are greatly reduced in size and number and the islet cells appear necrotic with marked vacuolations; (**3**) PC: cyto-architecture appears distorted. Langerhans islets are reduced in size and number, with necrotic acinar cells and degenerate islet cells; (**4**) TG1: Langerhans Islets appear to be small, with minor vacuolations and cyto-architecture distortion; (**5**) TG2: Langerhans Islets appear to be large and defined. Minor vacuolations and cyto-architecture distortion are present. Secretory acini, centroacinar cells and excretory ducts appear to be well defined; (**6**) TG3: Islet cells appear to be prominent and large. Edges seem to be well defined.

At the end of the 14 days, the animals were sacrificed and the pancreases removed. All pancreatic tissues were handled as described earlier and thoroughly examined (Figure 7). Compared to DC, Langerhans islets in the pancreatic tissues of the non-diabetic rats (NC) were nearly twice as large. The difference between the two groups was significant (*p <* 0.01). GLES at 1000 and 500 mg/kg b.w. caused 44% and 50% respective increases in the average area of Langerhans islets compared to DC. Compared with NC, GLES (500 and 1000 mg/kg) yielded Langerhans islets that were ~80% as large. This effect was not observed when metformin (500 mg/kg) was administered with the same frequency.

### 3.4. Effect of G. latifolium Extract on Muscle Glucose Uptake and Jejunum Glucose Absorption

Rat abdominal muscle segments were isolated, weighed and incubated at 37 °C. Glucose uptake by each segment was calculated as previously described. When introduced to the muscles *in vitro*, both GLES and insulin did not increase the uptake of glucose by much. However, when combined, the effect was more than each one alone, indicating mild insulin-sensitization activity (Table 3). To observe the effect of GLES on glucose absorption via the intestinal tract, rat jejunum sacs were prepared and calculations made as described earlier. Both Phlorizin and Acarbose were shown to reduce glucose active transport via rat jejunum sacs (Table 4). On the other hand, intestinal glucose transport was not influenced by GLES. Hence, it may be safely concluded that the decrease in postprandial BGL caused by the extract, as shown earlier in OGTT (Figure 1), did not result from delayed glucose absorption via the intestinal tract.

### 3.5. GC-MS Analysis of GLES

In this study, GC-MS analysis showed GLES to contain 15 volatile compounds with high certainty (Table 5). It was found to be mainly rich in saturated fatty acid esters, such as Methyl Palmitate, Methyl Stearate and Methyl Arachidate; and long chain hydrocarbons such as Nonacosane and Palmitic Acid. It also contained an unsaturated fatty acid ester, Methyl Oleate and an unsaturated fatty alcohol, linolenyl alcohol. Phytol, D:C-friedoolean-8-en-3-one and stigmast-4-en-3-one (sitostenone) were amongst the major volatile compounds found in GLES.

## 4. Discussion

*Gongronema latifolium* is an herbaceous shrub with yellow flowers [13] referred to as utazi and arokeke in South Eastern and South Western Nigeria, respectively [9]. It is widespread in the tropical and subtropical regions especially in Africa and South America, with a moderate representation in Northern and South Eastern Asia [32]. The LD_50_ of an extract of GL given by the intraperitoneal route was found to be 1678.63 ± 78 mg/kg b.w. in mice [33] and the oral LD_50_ was estimated to be greater than 5000 mg/kg b.w. in albino rats [34]. In the present study, the doses of GLES administered to the SD rats were between 500–2000 mg/kg b.w/day.

Rising longevity, consumption of calorie-rich diets, obesity and a sedentary lifestyle has led to a marked increase in the number of diabetics worldwide [35] and contributed to the growing interest among researchers to investigate the therapeutic potential of plants [36]. According to Pouwer and Hermanns [37], the management of diabetes should focus on three main targets: prevention of hyperglycemia and its associated complications, prevention of hypoglycemia and maintenance of the patient’s quality of life. This paper investigated the antihyperglycemic activity of *Gongronema latifolium* upon acute and subchronic treatments; and the results confirmed the antihyperglycemic activity observed earlier by Ugochukwu and Babady [23], but using a greater dose. This implied that Soxhlet extraction may have affected the antihyperglycemic potential of GL by way of lost heat-labile active principal(s). This is in contrast with the soxhlet extract of West African *Morinda lucida* leaves, whose hypoglycemic effect was shown to be superior to tolbutamide, a sulfonylurea, in diabetic rats at comparatively lower doses than GL in the present study [38]. However, with a complex disorder such as DM, achieving optimum blood glucose control is only half the solution towards achieving good quality of life. Currently available oral anti-diabetic drugs often lead to weight gain and cause an obese condition [39]. Furthermore, DM has been associated with dysregulation of food intake, probably due to impaired vagal outflow arising from hyperglycemia [40]. As observed in this present study, GLES had no negative impact on body weight and food intake rates in diabetic rats.

Dyslipidaemia is a common manifestation in DM cases and is associated with greater risk of atherosclerosis because of increased levels of triglycerides, VLDL and LDL, presence of small dense LDL particles and decreased HDL levels [41]. STZ-induced diabetic rats presented with elevated blood cholesterol and triglyceride levels [42]. In this study, the diabetic animals which received 1 g/kg of GLES sub-chronically developed high total cholesterol levels (TC), although they had close-to-normal TC/HDL ratios, also referred to as the Cardiac Risk Ratio (CRR) [43], indicating a clear elevation in the serum HDL levels. This activity of GLES could prove to be valuable in countering premature atherosclerosis associated with Type 2 DM. This proposition is in tandem with that of Brai*, et al.* [44] about the observed effect of leaf extracts of *Persea Americana* on lipid metabolism in hypercholesterolemic rats. On the other hand, as opposed to Ugochukwu, Babady, Cobourne and Gasset [9], GLES had no reducing effect on LDL levels in any of the treated groups. This lost effect perhaps signals lost heat-labile active principal(s) as the report cited above utilized a cold extraction method to prepare GL ethanolic extract. Lastly, in agreement with [45], reductions in the levels of TC and LDL caused by metformin were rather small. Present data also showed that daily doses of GLES as high as 1000 mg/kg b.w. had no significant effect on the levels of the renal function parameters tested.

The understanding of the mechanisms which underlie GL’s antihyperglycemic effect is still lacking. Possible mechanisms of action have been suggested. The effect was attributed to an insulin-like activity of the ethanolic extract [23] and more recently an insulinotropic activity [22]. Ogbu*, et al.* [46] attributed the decrease in BGLs partly to delayed gastric emptying. Our *in vitro* findings showed that GLES had no effect on glucose active transport via the intestinal wall. Perhaps, the insulin-sensitizing activity observed in this work might be one of the mechanisms by which the extract exerts its antihyperglycemic effect, especially because such a mechanism seems to be consistent with our findings on the level of serum insulin following the 14-day treatment. 

Langerhans islets are found in the exocrine acinar cells of the pancreas and are composed of several endocrine cells: α-cells, β-cells, δ-cells, and PP-cells [47]. β-cells constitute the major cell type (occupying 70%–80%) in an islet [48] and are responsible for insulin secretion in response to elevated BGLs. As indicated by our findings and in agreement with [7], unlike metformin, GLES was found to act directly on the pancreas to induce the regeneration of a tissue that appeared well structured and Langerhans islets that appeared well defined and larger in area compared with the diabetic control in a statistically non-significant manner. Nagappa*, et al.* [49] noted similar partial restoration of normal cellular population when investigating Indian Almond, *Terminalia catappa*. This probably translated into the observed higher serum insulin levels in TG3 compared with DC in this study. Nevertheless, the current results ought to be considered within the context of some limitations: Diabetes mellitus was induced in the animals by partial destruction of pancreatic cells, especially β-cells, using a dose of bacteria-derived Streptozotocin (STZ), a substance which is known to cause oxidative stress [50]. However, the antioxidant components present in GL extracts are so chemically diverse that both chlorophyll-enriched and chlorophyll-depleted fractions were found to possess considerable antioxidant properties [24]. Hence, what might have been perceived as restoration of Langerhans islet area could have been a result of GL’s established antioxidant potential [9,51] countering further oxidative damage caused by STZ. Furthermore, although our findings suggested that GLES increased the average area of Langerhans islets up to 50% compared to DC, Gomori stain remained insufficient to determine the viability of the β-cells within those islets. Further research is necessary to validate our conclusions using more advanced staining techniques of immunohistochemistry [27].

Over the past two decades, different parts of *Gongronema latifolium* have been found to contain saponins, anthraquinones, alkaloids, β-sistosterol, lupenyl esters, pregnance ester, glucosides and essential oils [10,52]. Indicating possible synergism between its components, the crude methanolic extract was reported to have a higher antihyperglycemic activity *in vivo* than its individual fractions and a comparable insulinotropic effect to glibenclamide [22]. Literature seems to indicate that GL acts to stimulate the secretion of insulin from the pancreas via non-phenolic molecules, as the methanol used by Fasakin, Udenigwe and Aluko [24] probably resulted in an extract that was poor in phenolic compounds. The active principles responsible for GL antidiabetic effect are yet to be fully characterized. In the current study, Stigmast-4-en-3-one, also known as Sitostenone, was found to be present in GLES. It is an active phytosterol which possesses an activity against NO release [53] and has been implicated in the hypoglycemic effects of a couple of herbal extracts [54,55]—possibly making it one of the chemical entities responsible for the moderate blood glucose lowering effect of GLES observed in this work. Some fatty acids such as those found in GL extracts have been strongly implicated in known anti-diabetic mechanisms [56]. Further research is still needed to elucidate the components of these extracts using more elaborate techniques like HPLC and IR.

## 5. Conclusions

*Gongronema latifolium* Benth. (Apocynaceae) displayed a moderate significant blood glucose lowering effect with high daily doses in sub-chronic treatment in the present study. Although the tested extract produced a significantly improved lipid profile and mild regenerative effect at the level of the pancreas, among other beneficial aspects, we speculate that Soxhlet extraction might have contributed to the loss of valuable heat-labile active principles from the plant. The activity retained by our extract however, should indicate the presence of heat-stable active principles as well, such as Sitostenone, a glucose lowering agent that we report as one of the major volatile compounds present in *G. latifolium*.

## Figures and Tables

**Table 1:** Animal Grouping: Acute treatment with *Gongronema latifolium* extract.

Group	Treatment
TG1	500 mg/kg b.w. of GLES
TG2	1000 mg/kg b.w. of GLES
TG3	2000 mg/kg b.w. of GLES
Diabetic Control (DC)	10 mL/kg b.w. of distilled water
Positive Control (PC)	500 mg/kg b.w. of metformin
Non-Diabetic Control (NC)	10 mL/kg b.w. of distilled water

**Table 2:** Animal Grouping: Sub-chronic treatment with *Gongronema latifolium* extract.

Group	Treatment
TG1	250 mg/kg b.w. of GLES
TG2	500 mg/kg b.w. of GLES
TG3	1000 mg/kg b.w. of GLES
Diabetic Control (DC)	10 mL/kg b.w. of distilled water
Positive Control (PC)	500 mg/kg b.w. of metformin
Non-Diabetic Control (NC)	10 mL/kg b.w. of distilled water

**Table 3:** Effect of an ethanolic extract of *Gongronema latifolium* on glucose uptake by isolated rat abdominal muscle incubated in the presence/absence of insulin (mg glucose/g tissue) (*n* = 5).

Control	Insulin (1 IU/mL)	GL (1 mg/mL)	Insulin + GL
285.2 ± 10.81	328.52 ± 9.43	319.87 ± 29.41	392.54 ± 34.16

**Table 4:** Effect of an ethanolic extract of *Gongronema latifolium* on glucose active transport via isolated rat jejunum (*n* = 6).

Control	Phlorizin (1 mg/mL)	Acarbose (1 mg/mL)	GL (1 mg/mL)
52.62 ± 13.66	22.32 ± 9.94	21.78 ± 6.87	50.58 ± 11.52

**Table 5:** GC-MS spectral analysis of ethanolic extract of *Gongronema latifolium*.

No.	RT (min)	Name of the Compound	Molecular Formula	Peak Area %	Quality
1	3.15	Triethyl borate	C_6_H_15_BO_3_	0.51	95
2	11.62	Methyl Palmitate	C_17_H_34_O_2_	11.28	98
3	11.87	Trichloroacetic acid, hexadecyl ester	C_18_H_33_Cl_3_O_2_	0.41	87
4	11.95	Ethyl Palmitate	C_18_H_36_O_2_	2.46	95
5	12.00	Palmitic acid	C_16_H_32_O_2_	2.52	94
	12.03			3.86	95
	12.10			5.71	95
6	12.43	Methyl Oleate	C_19_H_36_O_2_	5.32	99
7	12.46	Linolenyl alcohol	C_18_H_32_O	3.40	97
8	12.52	Methyl stearate	C_19_H_38_O_2_	2.53	99
9	13.01	Phytol	C_20_H_40_O	20.53	91
10	13.42	Methyl Arachidate	C_21_H_42_O_2_	1.53	97
11	14.30	Octadecanoic acid, 10-methyl-, methyl ester,(R)	C_20_H_40_O_2_	0.69	94
		(or) Methyl Behenate	C_23_H_46_O_2_		
12	16.66	Nonacosane	C_29_H_60_	5.48	98
13	18.82	Eicosane	C_20_H_42_	7.01	95
		(or) Hentriacontane	C_31_H_64_		
14	24.3	D:C-Friedoolean-8-en-3-one	C_30_H_48_O	12.35	87
15	26.41	Stigmast-4-en-3-one (Sitostenone)	C_29_H_48_O	6.64	98

## Abbreviations

The following abbreviations are used in this manuscript:

GL: *Gongronema latifolium*
GLES: *Gongronema latifolium *ethanolic Soxhlet extract
DM: Diabetes mellitus
STZ: Streptozotocin
FBG: Fasting blood glucose
BGLs: Blood glucose levels
b.w: Body weight
SD: *Sprague-Dawley*
GC-MS: Gas chromatography-mass spectrometry
NC: Normal control
DC: Diabetic control
PC: Positive control
TG: Treatment group
WHO: World Health Organization
OGTT: Oral glucose tolerance test
LDL: Low-density lipoproteins
HDL: High-density lipoproteins
TC: Total cholesterol
LD_50_: Median lethal dose
